# Predicting the Potential Distribution of the Invasive Plant *Alternanthera pungens* Kunth Under Climate Change Scenarios in China

**DOI:** 10.1002/ece3.73124

**Published:** 2026-02-24

**Authors:** Fengping Zheng, Wei Zhang, Qiurui Li, Zhijie Wang, Gaofeng Xu, David Roy Clements, Bin Yao, Guimei Jin, Shaosong Yang, Shicai Shen, Fudou Zhang, Michael Denny Day

**Affiliations:** ^1^ Key Laboratory of Prevention and Control of Biological Invasions, Ministry of Agriculture and Rural Affairs of China Agricultural Environment and Resource Research Institute, Yunnan Academy of Agricultural Sciences Kunming China; ^2^ Key Laboratory of Green Prevention and Control of Agricultural Transboundary Pests of Yunnan Province Agricultural Environment and Resource Research Institute, Yunnan Academy of Agricultural Sciences Kunming China; ^3^ Yunnan Lancang‐Mekong Agricultural Bio‐Security International Science and Technology Cooperation Joint Research Center Agricultural Environment and Resource Research Institute, Yunnan Academy of Agricultural Sciences Kunming China; ^4^ School of Ethnology and Sociology Minzu University of China Beijing China; ^5^ School of Agriculture Yunnan University Kunming China; ^6^ Department of Biology Trinity Western University Langley British Columbia Canada; ^7^ Department of Primary Industries Brisbane Australia

**Keywords:** climate change scenarios, invasive alien species, MaxEnt, potential distribution

## Abstract

*Alternanthera pungens* Kunth is considered to be less invasive compared to its exotic congener *A. philoxeroides* (Mart.) Griseb. However, in recent 10 years, it has spread rapidly in Yunnan Province, China. To better understand the species' invasion and distribution, we simulated the potential distribution of *A. pungens* in China using a MaxEnt model under the current climate scenario and several future climate scenarios, with varying emissions and time frames. The model achieved excellent prediction performance, with *A. pungens* having an area under the curve value and true skill statistics value of 0.979 and 0.910, respectively. Temperature seasonality and mean temperature of coldest quarter were the greatest predictive environmental variables, with a cumulative contribution of more than 85.3% and a cumulative permutation importance of more than 89.8%. The suitable geographic region of *A. pungens* is concentrated in southern China. Under the current climate scenarios, projected areas ranked as highly and moderately suitable for *A. pungens* accounted for 0.31% and 1.03% of the Chinese mainland area, respectively. Under future climate scenarios, the suitable areas for *A. pungens* in China will expand northwards, with a maximum projected growth rate of 41.4% in the 2070s. This study was the first to show that *A. pungens* is predicted to expand its range in China in the future. Early warning and monitoring of *A. pungens* should be pursued, with greater vigilance in southern China to prevent its further spread and invasion.

## Graphical abstract

The potential distribution of *A. pungens* in China was simulated using a MaxEnt model under current and future climate scenarios.

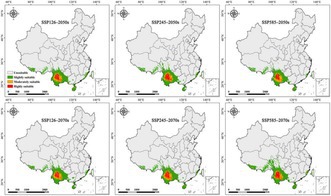

## 1 Introduction

Global climate change and biological invasions have continually gained significant attention because they exert unprecedented pressure on agricultural ecosystems and natural environments worldwide (Early et al. 2016; Bellard et al. 2017; Pyšek et al. 2020). Biological invasions are a growing integrated component of global environmental change as international trade, economic industrialization and human activities increase (Pyšek et al. 2012; Simberloff et al. 2013; Diagne et al. 2021). Invasive alien species are recognized as a main driver of global biodiversity loss and cause significant negative ecological, economic, and social consequences (Rai and Singh 2020; Hudgins et al. 2023; Peller and Altermatt 2024; Shen et al. 2024).

Climate change is considered a primary cause of biodiversity loss and is aiding the acceleration of the invasion and spread of invasive alien species (Thomas et al. 2004; Rahel and Olden 2008; Essl et al. 2020). Moreover, human‐mediated movement of species has broken down geographical and environmental obstacles, resulting in range expansion and an increase in risk in the invasion of invasive species (Seebens et al. 2015; Laginhas and Bradley 2022). The intricate interplay between climate change and invasive species is predicted to alter the distribution range of invasive species across the globe (Ren et al. 2025). Therefore, studying the responses of invasive species to climate change is important to learn of the future suitable geographical distribution and provides theoretical support and reference for prevention and management strategies of these species.

*Alternanthera pungens* Kunth (Amaranthaceae), commonly known as khaki weed, is a perennial creeping herb, native to Central and South America (Chen et al. 1993; Parsons and Cuthbertson 2001; CABI 2021). This species is widely distributed across temperate, tropical, and subtropical regions globally, encompassing countries such as Australia, Bhutan, China, India, Myanmar, Thailand, and the USA (Chen et al. 1993; Parsons and Cuthbertson 2001; Wang et al. 2010; Hephner et al. 2013; Jakhar and Dahiya 2017). *Alternanthera pungens* has been described as one of the fastest‐growing weed problems in semi‐arid and arid environments (Kopec et al. 2004). Due to its strong ecological and morphological adaptability, *A. pungens* can invade greatly varying habitats and soil types, such as brown soil, bauxite, semi‐arid areas, plains, and arid and hot valleys (Hephner et al. 2013; Jakhar and Dahiya 2017). *Alternanthera pungens* has vigorous growth, a deep taproot, and multiple means of reproduction, which makes it difficult to control (McEachin et al. 2024).

*Alternanthera pungens* is usually considered an unpleasant weed by local people because the spiny burrs on its leaves and the spines on the fruit can injure people and animals. Plants can also cause hay fever, asthma, and dermatitis, and skin ailments (Parsons and Cuthbertson 2001) and are believed to be poisonous to animals. Therefore, plants are not readily eaten by livestock, although young plants are sometimes consumed by sheep (Parsons and Cuthbertson 2001).

*Alternanthera pungens* can easily invade a variety of habitats by their spiny fruits attaching to animals, equipment, clothing, rubber‐soled shoes, and car tyres (Parsons and Cuthbertson 2001). Seeds are dispersed either as a prickly burr of many fruit or as individual spiny fruit. The strong stems and deep taproots enable plants to survive extreme conditions, and new plants can easily become established if stem or root fragments get adequate moisture (Parsons and Cuthbertson 2001).

In China, *A. pungens* was first reported in Lushan County of Sichuan Province in 1957, and is now widely present in southwestern Sichuan, Yunnan, Hainan, Guangdong, Hong Kong, and southern Fujian (Chen et al. 1993; Wang et al. 2010; Zeng et al. 2011; Shen et al. 2012). During a survey of invasive alien plant species in 2021–2022, *A. pungens* was found to widely distributed in Dali City, Kunming City, Baoshan City, Dehong Prefecture, Xishuangbanna Prefecture, Honghe Prefecture, Lijiang City, Diqing Prefecture, and other areas of Yunnan Province, China.

Compared to its exotic congener *A. philoxeroides* (Mart.) Griseb., *A. pungens* has been historically considered a relatively weak invasive species for a long time, due to its shorter invasion history, lower growth rate, smaller distribution area, and lesser economic impacts in China (Wang et al. 2010). Hence, research on the biology, ecology, prevention and control of *A. pungens* has been limited. However, recent observations suggest this assessment of *A. pungens* being a weak invasive species may need revision.

In China, the current distribution of *A. pungens* is limited to the southern provinces. However, due to its ability to produce many seeds that can spread long distances and the potential impacts of the weed, it is desirable to predict its potential distribution and expansion range in China for its future sustainable management.

Ecological niche models (ENMs) are quantitative tools designed to estimate the suitable environmental conditions for a species by modeling the relationship between known occurrence data and relevant ecological variables (Sillero 2011; Melo‐Merino et al. 2020). These models rely on the fundamental ecological principle that a species' geographic distribution is determined by its ecological niche, including both abiotic and biotic factors (Guisan and Thuiller 2005; Hirzel and Le Lay 2008). A variety of ENMs have been developed over past decades, including the Genetic Algorithm for Rule‐set Production (GARP) (Stockwell 1999), Bioclimatic Envelope Model (BIOCLIM) (Booth et al. 2014), Environmental Envelope DOMAIN Model (DOMAIN) (Carpenter et al. 1993), and the Maximum Entropy Model (MaxEnt) (Phillips et al. 2006).

Among these, the MaxEnt model is currently the most widely applied, due to its high predictive accuracy, capacity to handle presence‐only data, and tolerance of small sample sizes (Phillips and Dudík 2008). It employs a machine‐learning algorithm based on the principle of maximum entropy to estimate the probability distribution of a species' presence that is most spread out (i.e., closest to uniform) while constrained by the environmental characteristics of known locations (Elith et al. 2011; Radosavljevic et al. 2014). The model has been extensively applied in various fields such as biodiversity conservation (He et al. 2021; Li et al. 2022), species reintroduction planning (Harman et al. 2025; Zhang et al. 2025), disease vector modeling (Pramanik et al. 2021; Ma et al. 2023), as well as in predicting the spread and potential distribution of invasive species. In invasion biology, it has proven particularly valuable for forecasting the potential distribution of invasive plants under current and future climate scenarios (Yan et al. 2020; Shen et al. 2024; Wu et al. 2024), identifying areas at high risk of invasion, and informing early warning and rapid response strategies. The MaxEnt model is useful to predict the future potential suitable areas in terms of the biology and ecology of the study species.

The objectives of the current study were to explore the potential distribution of *A. pungens* using the species distribution model MaxEnt under current and future climate scenarios in China, and provide useful information to help minimize and mitigate invasion and spread by *A. pungens* .

## 2 Materials and Methods

### 2.1 Species Occurrence Records Collection

**FIGURE 1:**
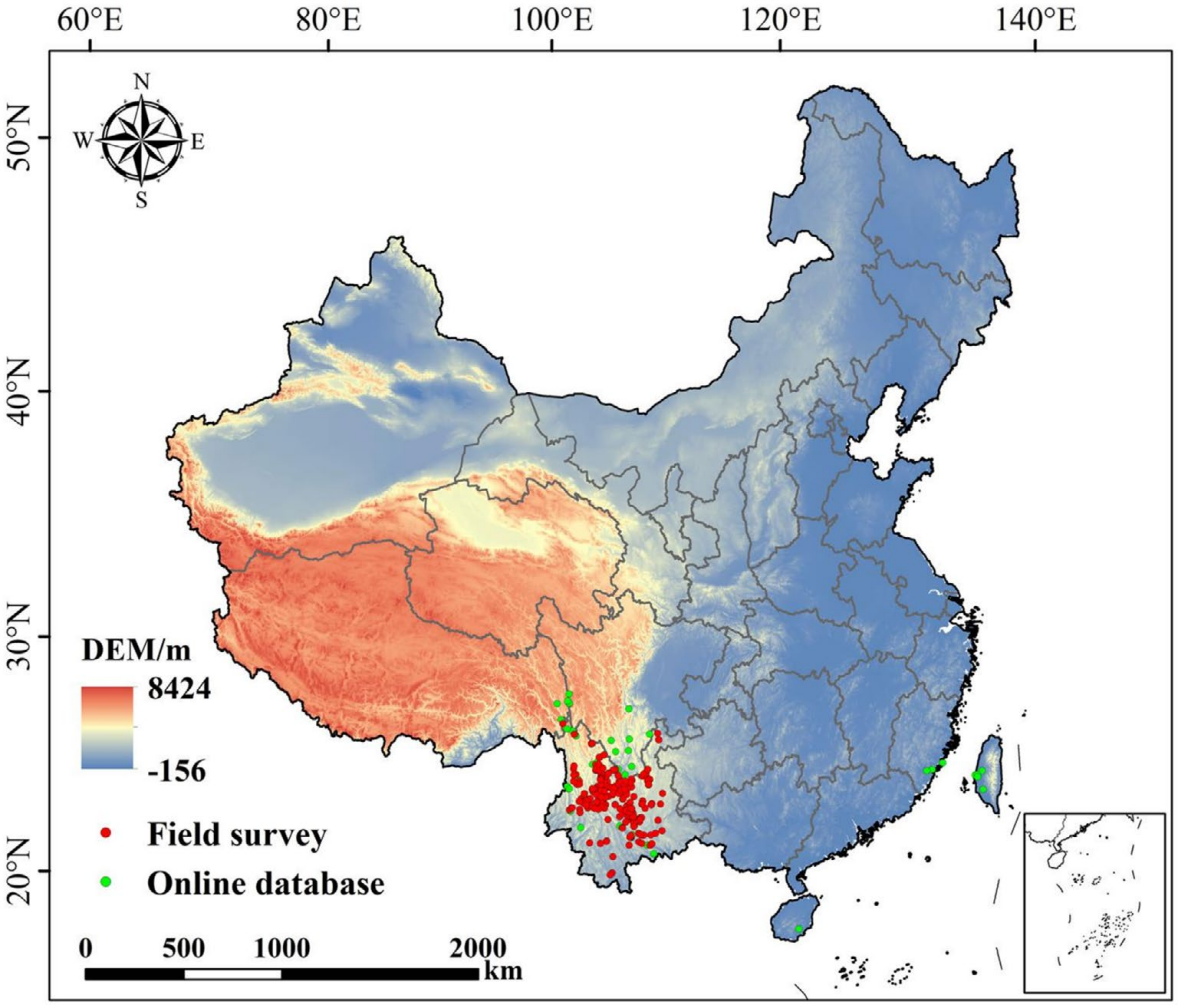
Distribution of sampling sites for *Alternanthera pungens* in China.

A total of 372 occurrence records of *A. pungens* in China were obtained using a Global Positioning System (GPS) during field surveys. In addition, 46 records were acquired from online databases, including the Global Biodiversity Information Facility (GBIF, https://www.gbif.org/), the Chinese Virtual Herbarium (CVH, https://www.cvh.ac.cn/), and the National Specimen Information Infrastructure (NSII, http://www.nsii.org.cn/). Based on these data, a map of the recorded distribution of *A. pungens* in China was generated (Figure 1).

To reduce spatial sampling bias and minimize pseudo‐replication, we applied the “remove duplicate occurrences” function in ENMtools (Warren et al. 2010), retaining only one record per 2.5′ × 2.5′ grid cell. After filtering, a total of 276 occurrence record points of the original 418 records were retained for MaxEnt modeling.

### 2.2 Environmental Variables Selection

Environmental variable data were downloaded from WorldClim (https://worldclim.org/), including one topographic variable (elevation) and 19 bioclimatic variables across three time periods: the current period (1970–2000), the 2050s (2041–2060), and the 2070s (2061–2080). Future bioclimatic data were derived from the Beijing Climate Center Climate System Model (BCC‐CSM2‐MR) under the sixth phase of the Coupled Model Intercomparison Project (CMIP6). Three Shared Socio‐economic Pathways (SSPs) were selected to represent different greenhouse gas emission scenarios: SSP126 (low), SSP245 (moderate), and SSP585 (high). All environmental variables were at a spatial resolution of 2.5 arc‐minutes.

**FIGURE 2:**
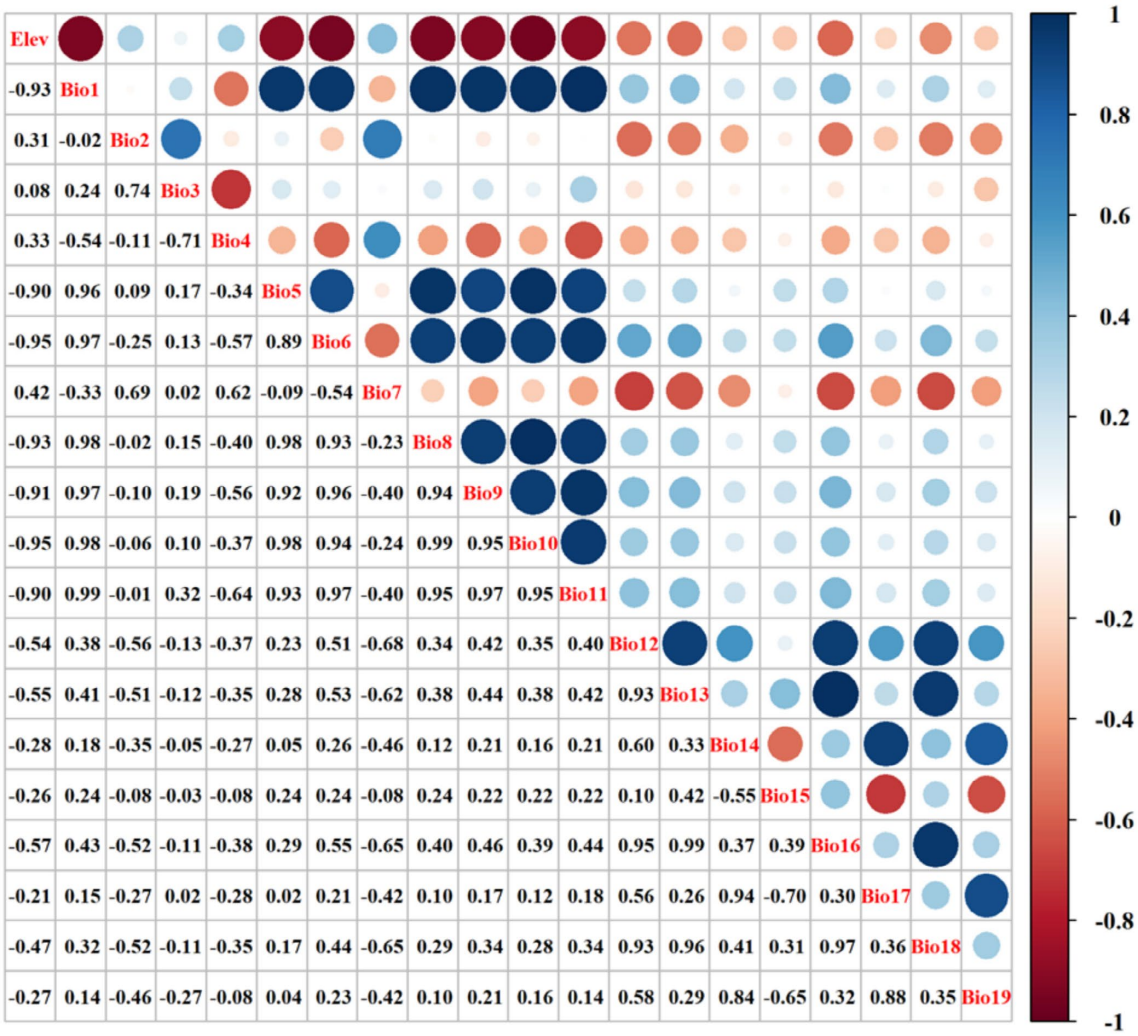
Correlation test for 20 environmental variables.

To reduce model overfitting and improve prediction accuracy by addressing multicollinearity among variables (Stuhldreher and Fartmann 2018), we conducted a Pearson correlation analysis on the 20 environmental variables using SPSS 27 (Figure 2). Variables with a high degree of correlation (|*r*| > 0.7) and low contribution, as evaluated by the default MaxEnt variable contribution analysis (Table 1), were excluded. Ultimately, five environmental variables were selected for use in the final model (Table 2).

**TABLE 1:** Contribution rate and permutation importance of 20 environmental variables.

Environmental variable	Contribution rate/%	Permutation importance/%
Bio4	56.1	6.6
Bio3	22.8	38.3
Bio7	7.2	3.8
Bio12	3.3	1.6
Bio13	2.8	1.3
Bio11	1.5	1.3
Bio9	1.1	1.3
Bio6	1.1	3.9
Bio17	0.7	6.6
Bio16	0.7	4.5
Bio2	0.6	1.2
Bio5	0.6	1.8
Bio1	0.4	1.3
Bio15	0.4	0.8
Bio18	0.3	16.5
Bio19	0.2	1.5
Bio10	0.1	0.2
Bio14	0.1	0.7
Bio8	0.1	0.4
Elev	0.1	6.6

**TABLE 2:** Environmental variables used in the model prediction for *Alternanthera pungens* .

Field	Description	Unit
Bio4	Temperature seasonality (standard deviation × 100)	—
Bio7	Temperature annual range	°C
Bio11	Mean temperature of coldest quarter	°C
Bio12	Annual precipitation	mm
Bio17	Precipitation of driest quarter	mm

### 2.3 Parameter Optimization of the MaxEnt Model

The predictive performance of the MaxEnt model is primarily influenced by two key parameters: Feature Classes (FC) and the Regularization Multiplier (RM) (Elith et al. 2011; Radosavljevic et al. 2014). Given the model's sensitivity to sampling bias and its tendency to overfit, using default parameter settings may result in unreliable predictions (Merow et al. 2013; Yan et al. 2021; Zhang et al. 2025). Therefore, we employed the R package kuenm (Cobos et al. 2019) to optimize model parameters.

The optimization process involved generating candidate models based on various combinations of FCs and RMs, evaluating their performance, and selecting the optimal configuration. Model selection followed a three‐step procedure: (1) identifying statistically significant models, (2) applying an omission rate threshold, and (3) selecting models with a statistically significant performance, an omission rate below 5%, and a Delta AICc ≤ 2 (Cobos et al. 2019). A total of 1160 candidate models were generated using 29 combinations of five FCs—linear (L), quadratic (Q), product (P), threshold (T), and hinge (H)—namely: L, Q, P, T, H, LQ, LP, LT, LH, QP, QT, QH, PT, PH, TH, LQP, LQT, LQH, LPT, LPH, QPT, QPH, QTH, PTH, LQPT, LQPH, LQTH, LPTH, and LQPTH; and 40 RM values ranging from 0.1 to 4.0, in increments of 0.1. After evaluating all candidate models, the configuration with a Delta AICc value of 0 was selected as the optimal parameter set for MaxEnt modeling (Cobos et al. 2019; Li et al. 2024; Miao et al. 2024).

### 2.4 Model Building and Evaluation

The 276 occurrence records of *A. pungens* and the five selected environmental variables were imported into MaxEnt v3.4.4, with the model configured using the previously optimized parameters. A random partitioning strategy was applied with 75% of the occurrence points used for model training and the remaining 25% occurrence points reserved for testing and validation. The maximum number of background points was set to 10,000, and the maximum number of iterations was limited to 500. The modeling procedure was repeated 10 times, and the final output was obtained by averaging the results across all replicates to ensure robustness. To assess the relative importance of each environmental variable and identify the key factors influencing the geographic distribution of *A. pungens* , we employed the Jackknife test embedded in MaxEnt.

Model performance was evaluated using the area under the curve (AUC) of the receiver operating characteristic (ROC) curve, which reflects the model's sensitivity (true positive rate) and specificity (false positive rate) (Bowers and Zhou 2019; Gebrewahid et al. 2020). An average AUC value between 0.8 and 0.9 indicates good model performance, while values exceeding 0.9 suggest excellent predictive capability (Sun et al. 2020; Luu et al. 2021). In addition, we used the true skill statistic (TSS), a metric significantly correlated with AUC, as a complementary performance measure (Allouche et al. 2006). TSS values between 0.6 and 0.8 denote good performance, and values above 0.8 indicate excellent model performance (Gama et al. 2017).

### 2.5 Classification of Suitable Areas

The ASCII output data generated by the MaxEnt model were imported into ArcGIS v10.4 and converted into raster format. To classify habitat suitability, we applied the maximum test sensitivity plus specificity (MTSPS) threshold, which is widely regarded as a conservative and ecologically meaningful criterion for species distribution modeling (Zhao et al. 2022). Specifically, areas with predicted suitability values below the MTSPS threshold were classified as unsuitable for species occurrence. Areas with values equal to or above the threshold were considered suitable and further classified into three levels of increasing habitat suitability (Li et al. 2019; Xie, Huang, Chen, et al. 2024). Based on this approach, the potential distribution of *A. pungens* in China was categorized into four classes for mapping and analysis purposes: unsuitable (0 ≤ *p* ≤ 0.0592), slightly suitable (0.0592 < *p* ≤ 0.4), moderately suitable (0.4 < *p* ≤ 0.6), highly suitable (0.6 < *p* ≤ 1).

### 2.6 Changes in Distribution and Centroid of Potential Suitable Areas

The raster outputs of suitable areas for *A. pungens* under different climate scenarios were classified into suitable and unsuitable areas using the MTSPS threshold of 0.0592, resulting in binary suitability maps. Using SDMtoolbox (Brown et al. 2017), a Python‐based GIS toolkit integrated with ArcGIS, we analyzed changes in the geographic extent of suitable areas and quantified the direction and distance of centroid shifts for *A. pungens* under current and future climate scenarios.

## 3 Results

### 3.1 Optimal Model and Accuracy Evaluation

**FIGURE 3:**
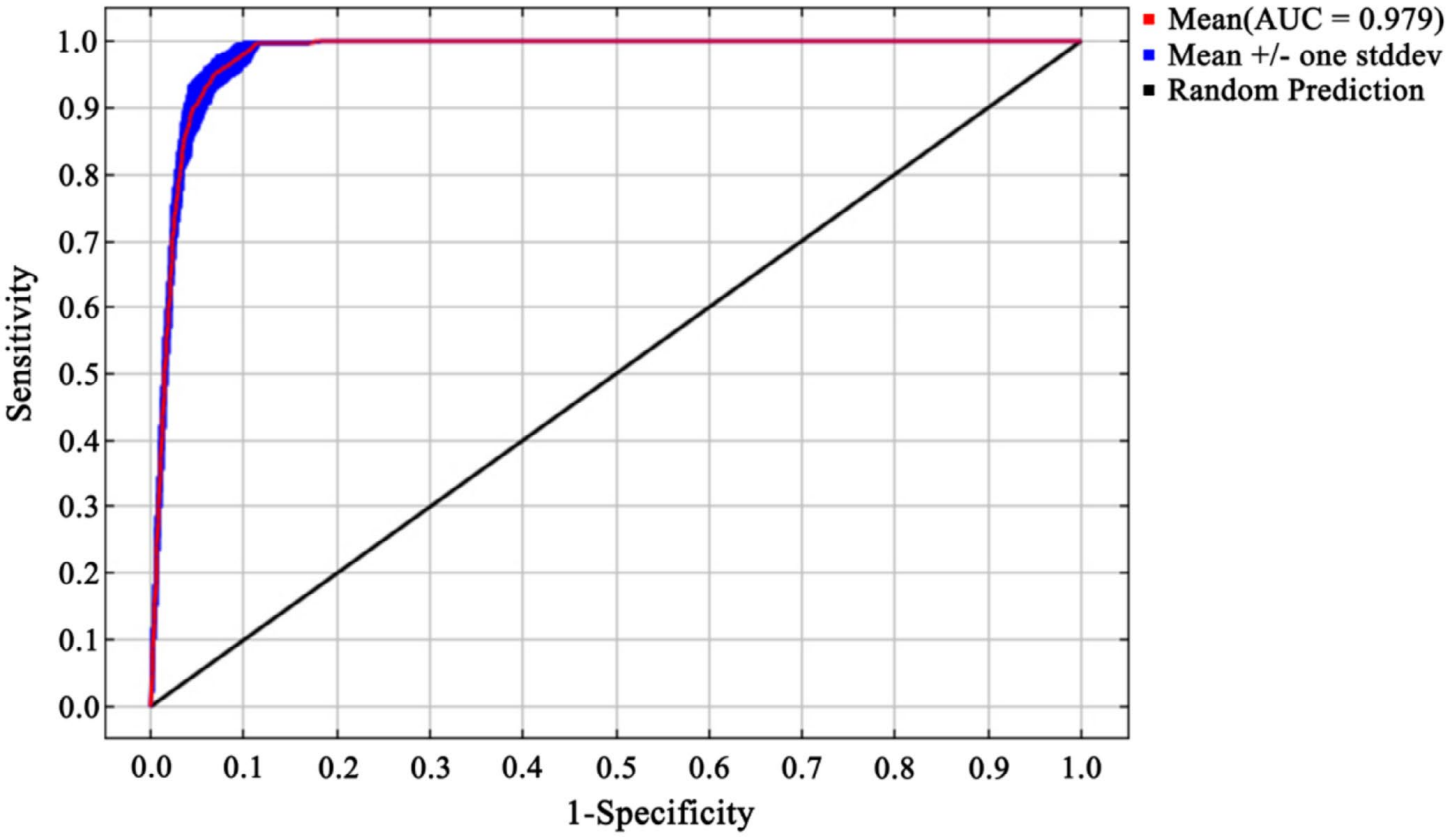
ROC test of the MaxEnt model with optimized parameters.

The default parameters (FC = LQPTH and RM = 1) of the model yielded a Delta AICc value of 30.29 (Table 3). The optimized model with parameters FC = LPT and RM = 1.3 achieved the lowest Delta AICc of 0, indicating the best model performance. Under this optimized configuration, the omission rate was reduced by 25.9%, suggesting an improved fit to the known occurrence data. Model evaluation using the optimized parameters produced a mean AUC of 0.979 ± 0.003 (Figure 3) and a mean TSS of 0.910 ± 0.018 (Table 4), demonstrating a high predictive accuracy and robustness of the model.

**TABLE 3:** Evaluation results of the MaxEnt model under different parameter settings.

Setting	FC	RM	Delta AICc	Omission rate
Default	LQPTH	1	30.29	0.058
Optimized	LPT	1.3	0	0.043

**TABLE 4:** TSS value of the MaxEnt model from 10 replicates with optimized parameters.

Models	TSS value
Species_0	0.876
Species_1	0.937
Species_2	0.917
Species_3	0.892
Species_4	0.916
Species_5	0.898
Species_6	0.912
Species_7	0.928
Species_8	0.919
Species_9	0.902
Mean	0.910

### 3.2 Key Environmental Variables Influencing the Distribution of *A. pungens*

**FIGURE 4:**
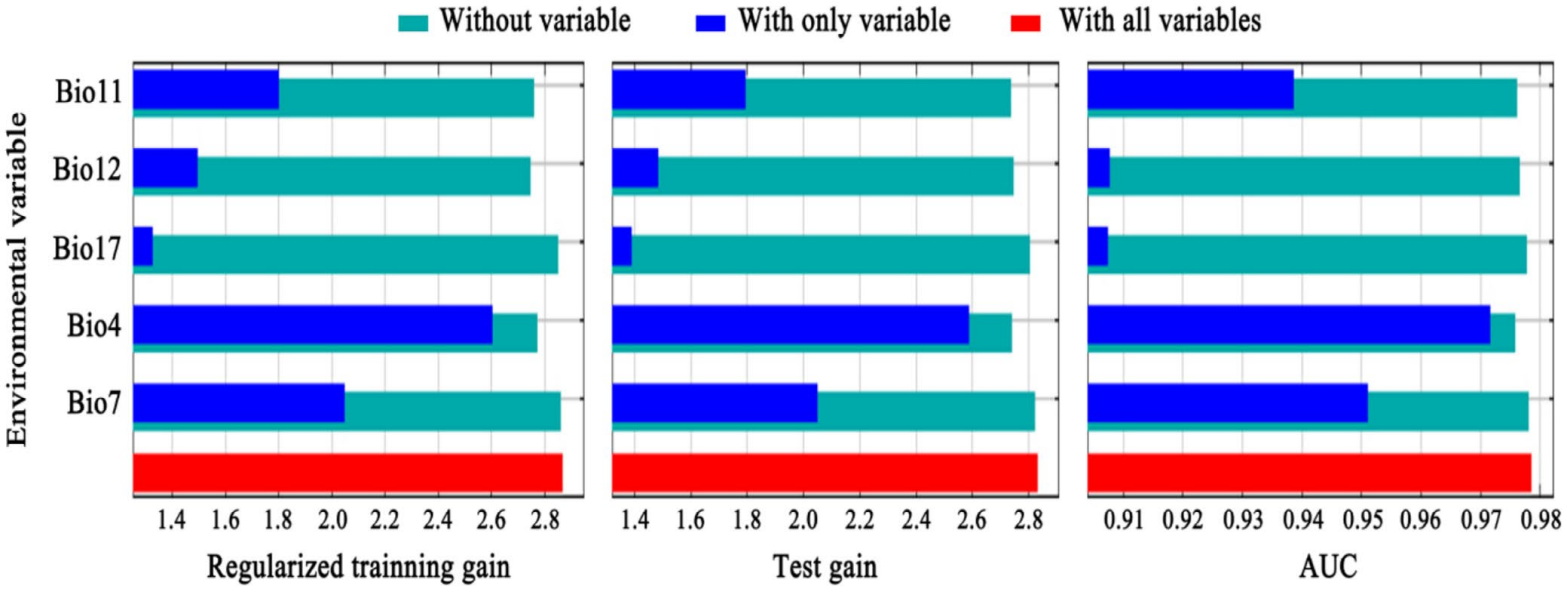
Jackknife test of environmental variables.

According to the Jackknife test (Figure 4) and the contribution rate and permutation importance results (Table 5), temperature seasonality (Bio4) was identified as the most influential variable among the five environmental factors used to construct the model. It had the highest contribution rate (80.4%) and permutation importance (54.3%), indicating its dominant role in determining the potential distribution of *A. pungens* . Mean temperature of coldest quarter (Bio11) also showed relatively high permutation importance (35.5%), although its overall contribution rate was limited (4.9%).

**TABLE 5:** Contribution rate and permutation importance of the five environmental variables used to construct the model.

Environmental variable	Contribution rate/%	Permutation importance/%
Bio4	80.4	54.3
Bio12	8.4	9.2
Bio7	5.5	0.4
Bio11	4.9	35.5
Bio17	0.8	0.7

**FIGURE 5:**
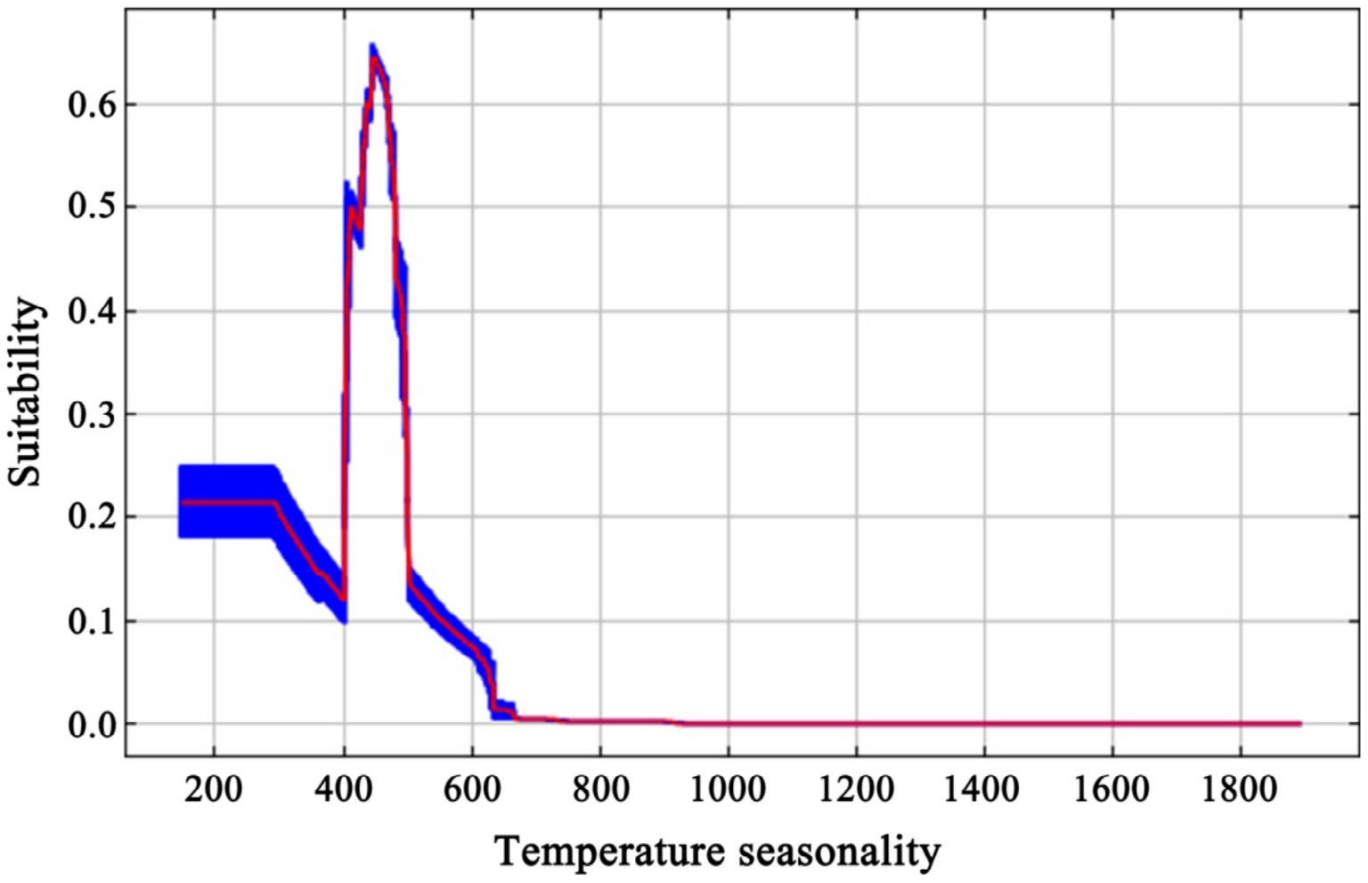
Area suitability response curve to temperature seasonality (Bio4).

The response curve of temperature seasonality (Figure 5) indicates that *A. pungens* would thrive in areas with values ranging from approximately 428 to 478, corresponding to a standard deviation of 4.28°C–4.78°C in mean monthly temperatures, where suitability values remain consistently above the 0.5 threshold. A suitability value exceeding 0.5 is commonly regarded as the threshold for favorable species occurrence (Huang et al. 2020). This suggests that the species favors regions with moderate temperature variability, while both low and high temperature seasonality reduce its suitability.

### 3.3 The Potential Distribution of *A. pungens* Under Different Climate Scenarios in China

**FIGURE 6:**
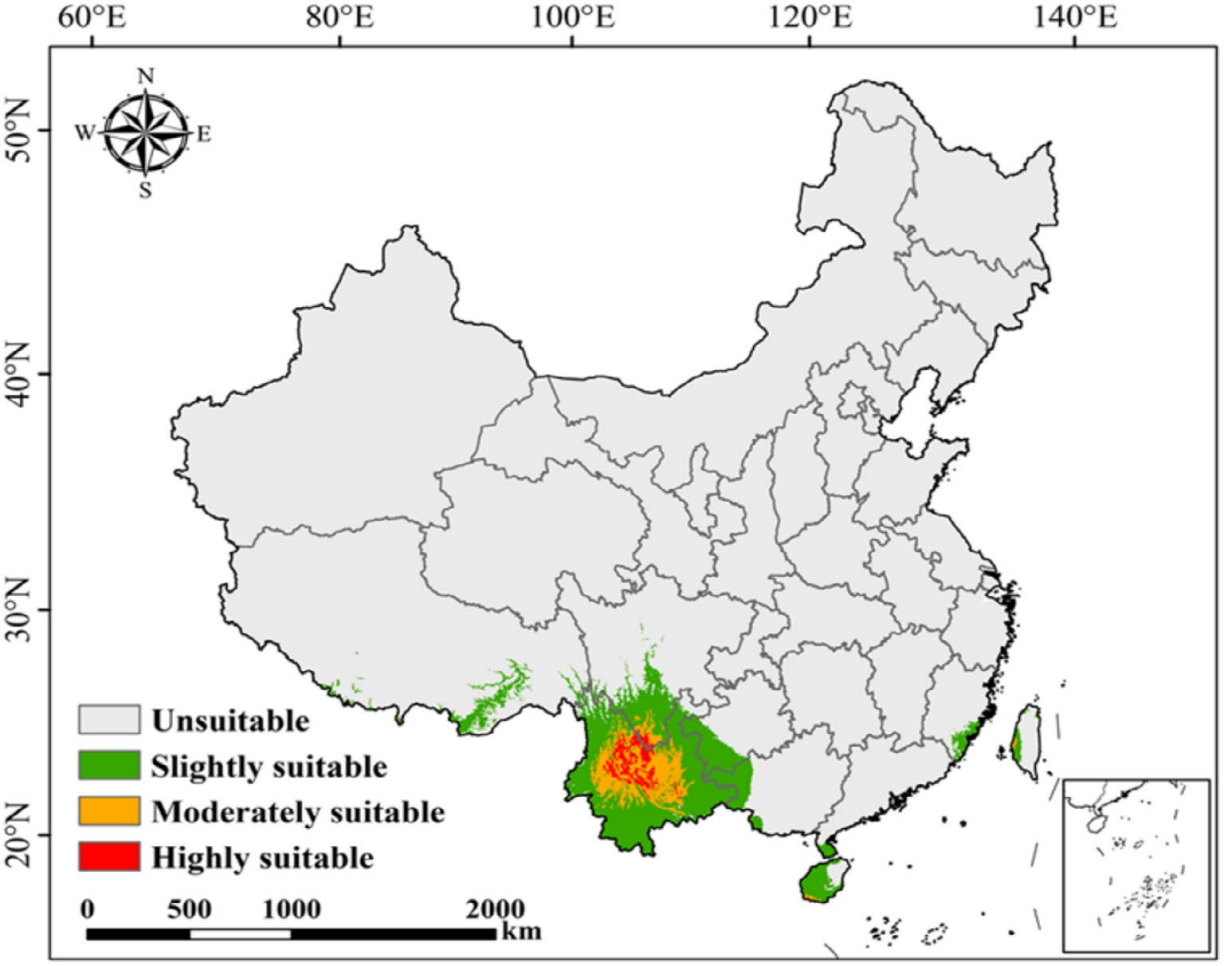
Geographic distribution of potential suitable areas for *Alternanthera pungens* under the current climatic conditions in China.

**FIGURE 7:**
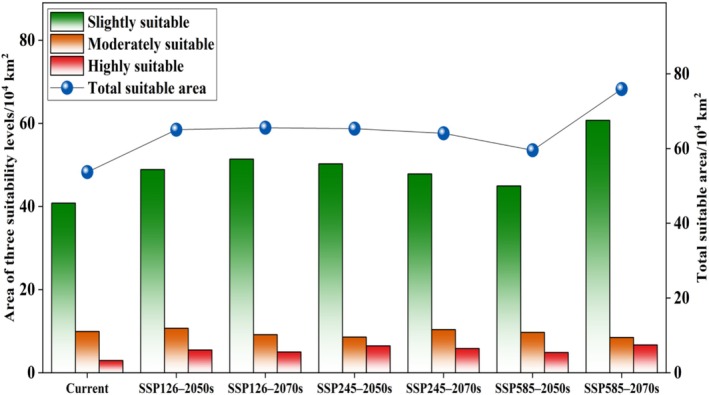
Area of potential distribution for *Alternanthera pungens* under different climate scenarios.

Under the current climatic conditions, the potential suitable areas of *A. pungens* are primarily concentrated in southwestern China (Figure 6), with a total suitable area of 53.66 × 10^4^ km^2^, accounting for 5.6% of China's land area (Figure 7). Among this, the highly suitable area covers 2.96 × 10^4^ km^2^ (5.6% of the total suitable area), mainly distributed in central Yunnan and southern Sichuan provinces. The moderately suitable area accounts for 9.9 × 10^4^ km^2^ (18.5%), while the slightly suitable area is the largest, covering 40.8 × 10^4^ km^2^ (76.0%).

**FIGURE 8:**
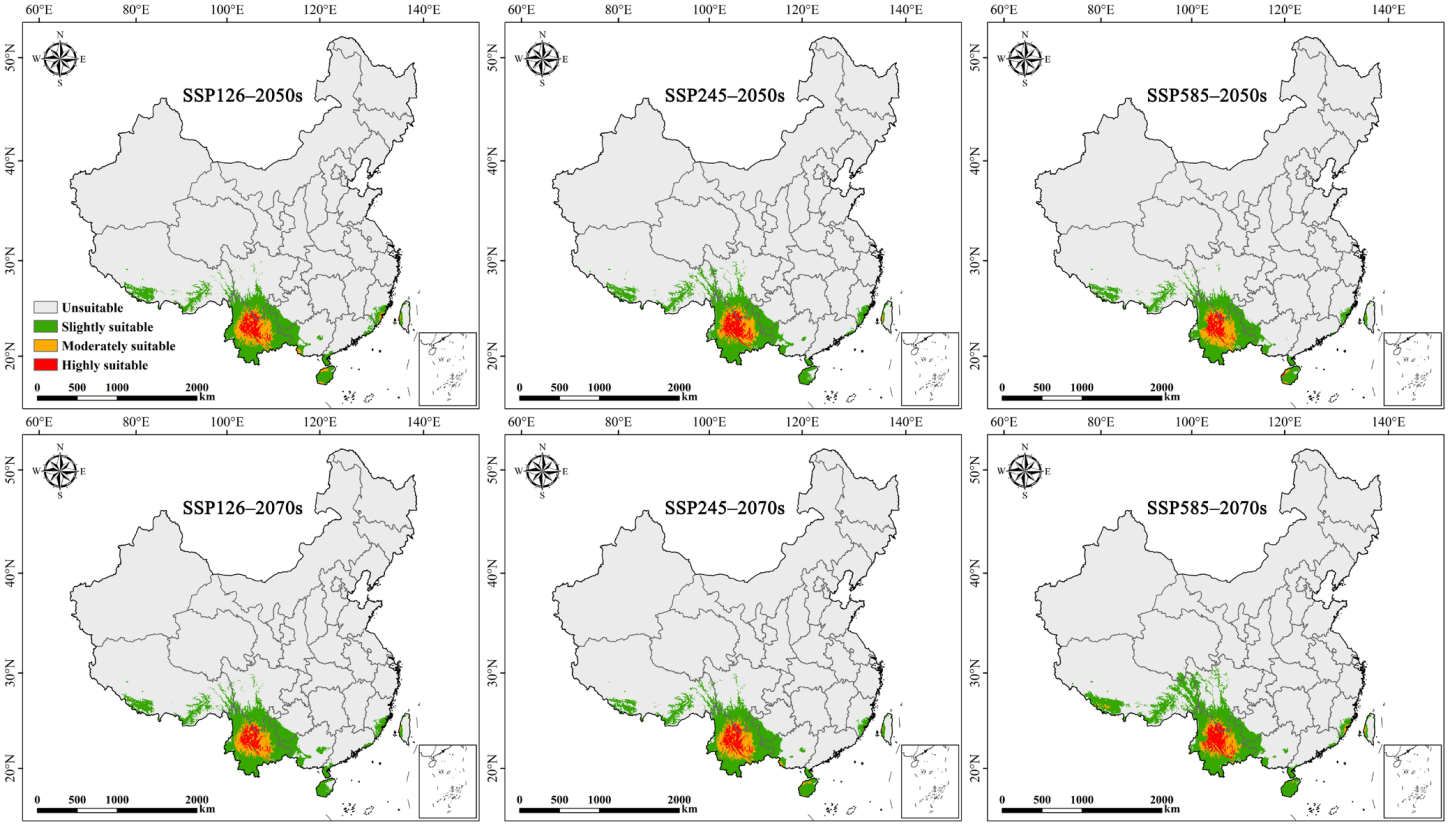
Geographic distribution of potential suitable areas for *Alternanthera pungens* under future climate scenarios in China.

**FIGURE 9:**
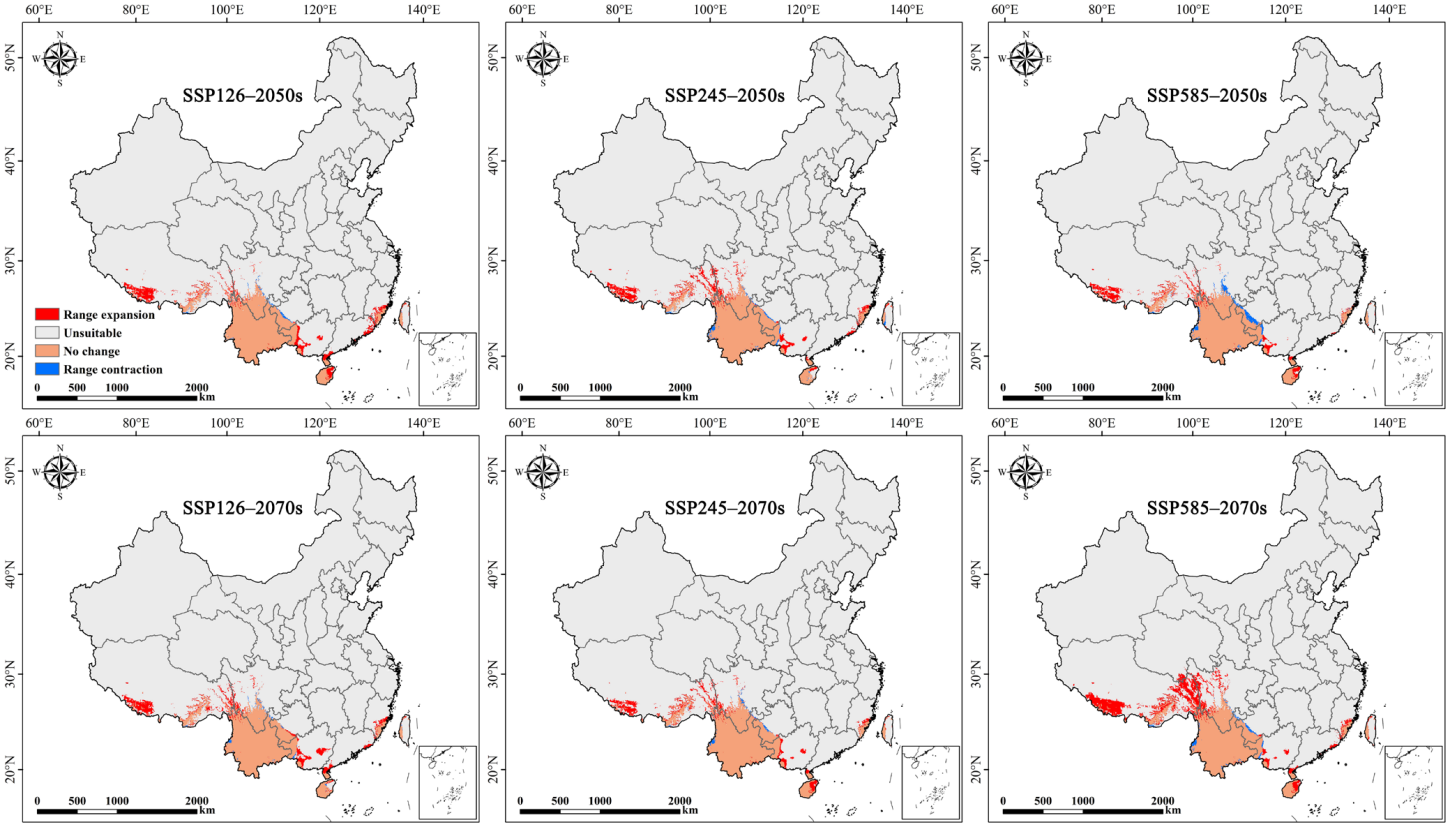
Changes in the geographic distribution of potential suitable areas for *Alternanthera pungens* under future climate scenarios in China.

**FIGURE 10:**
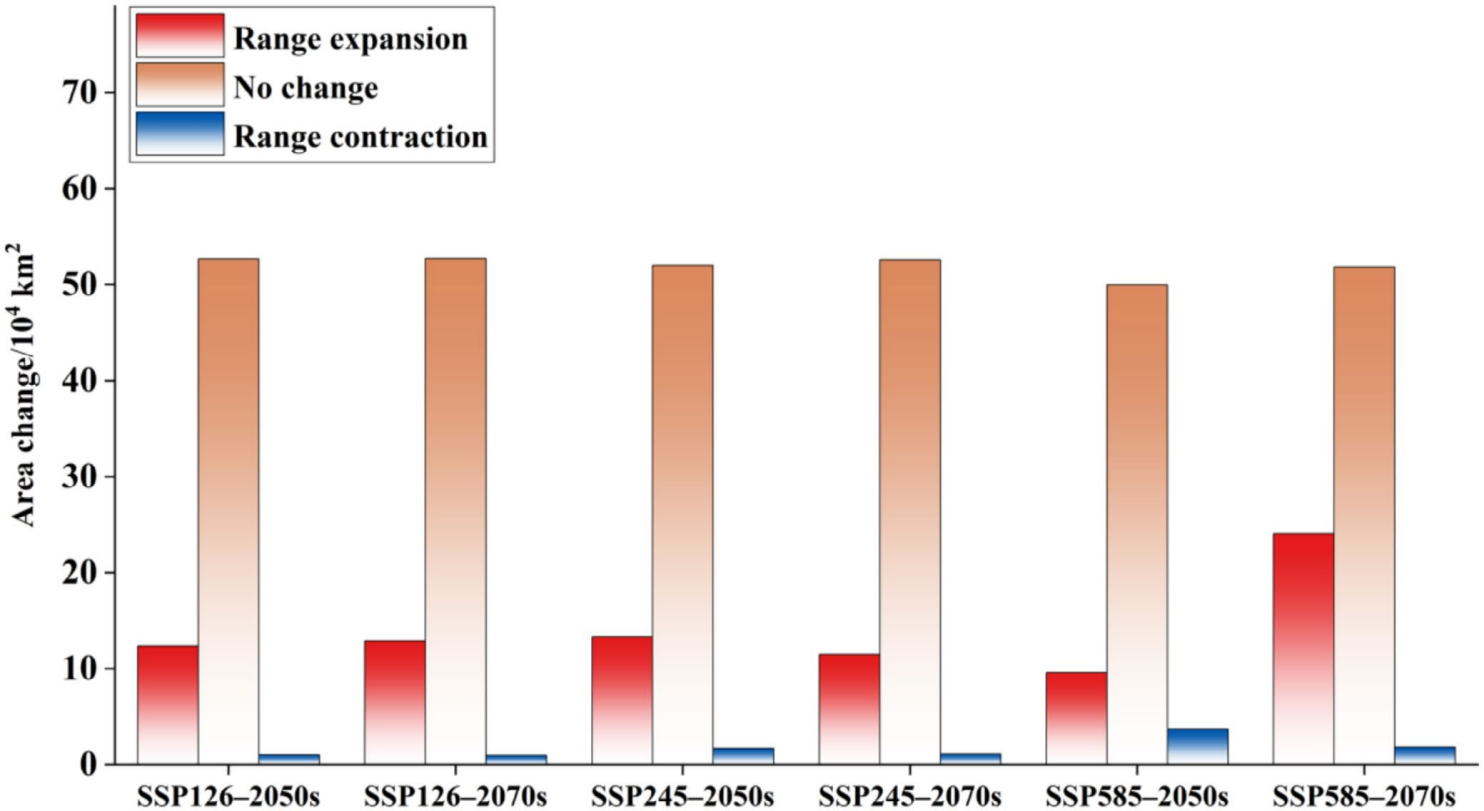
Area of potential distribution change for *Alternanthera pungens* under future climate scenarios.

Compared to the current climate, the suitable areas for *A. pungens* are projected to expand under all future climate scenarios (Figures 8 and 9). Under SSP126 and SSP245, the total suitable area is projected to increase modestly by approximately 19%–22% by the 2050s and 2070s. In contrast, under the high‐emission SSP585 scenario, the suitable area expands by only 10.9% in the 2050s but increases dramatically by 41.4% in the 2070s, reaching 75.9 × 10^4^ km^2^. Notably, certain suitable areas remain consistently present and geographically stable across all scenarios (Figure 10), suggesting strong climatic resilience. However, under SSP585 in the 2070s, a substantial net expansion of 22.2 × 10^4^ km^2^ occurs, whereas the 2050s show the lowest expansion (9.6 × 10^4^ km^2^) and the highest habitat contraction (3.7 × 10^4^ km^2^), indicating possible temporal instability in response to rapid climate change.

### 3.4 Centroid Shift of Potential Suitable Areas for *A. pungens*

**FIGURE 11:**
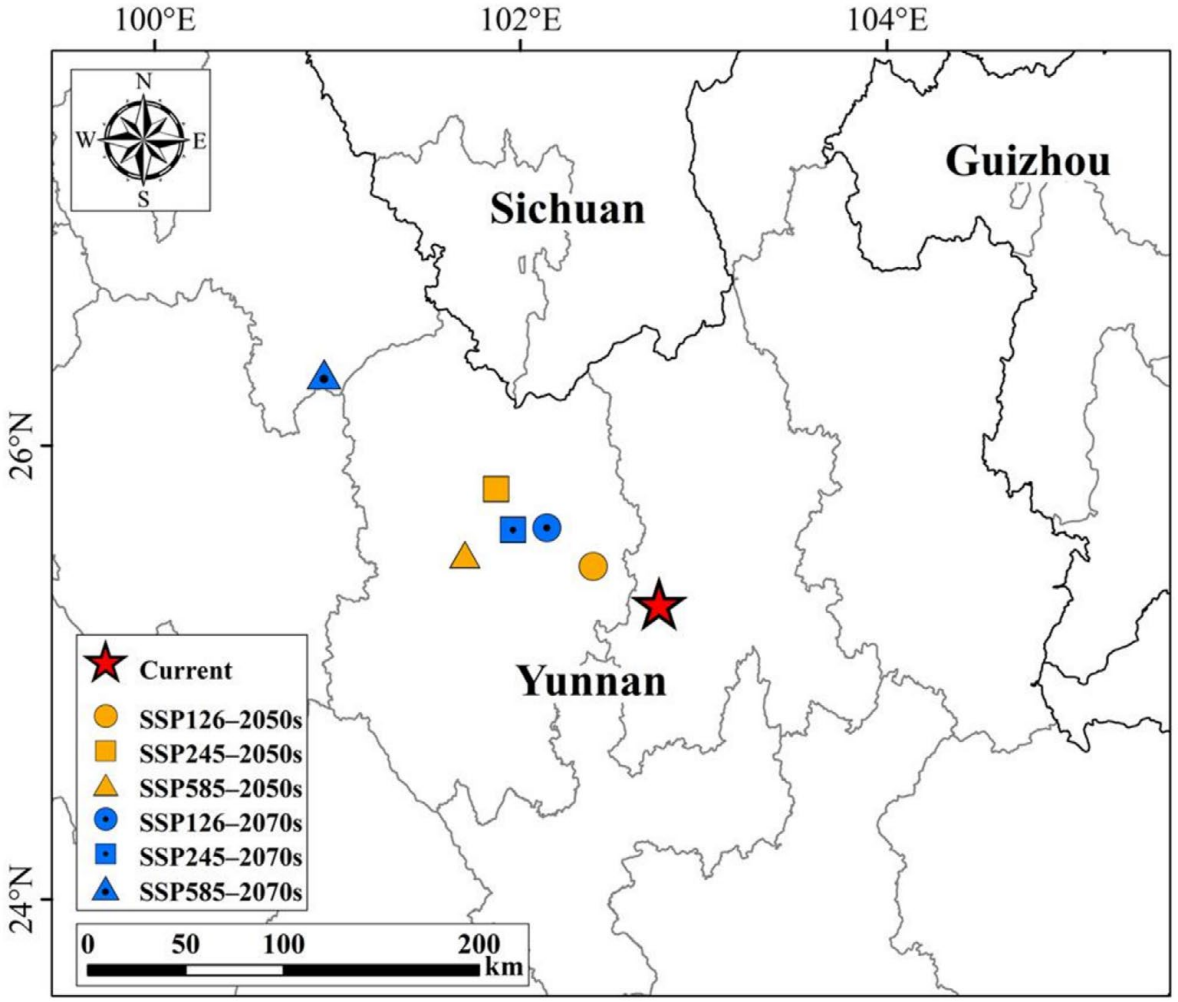
Centroid migration of *Alternanthera pungens* under future climate scenarios in China.

Although the migration patterns of the potential suitable area centroid for *A. pungens* vary under different climate scenarios, the overall trend consistently shows a northwestward shift (Figure 11). Under SSP126, the centroid gradually moves northwest by a total of approximately 70 km from the current position. In contrast, SSP245 shows a larger initial displacement of over 100 km to the northwest, followed by a slight southeastward correction. The most significant shift occurs under SSP585, where the centroid advances more than 220 km to the northwest by the 2070s. These results suggest that *A. pungens* is likely to expand into higher latitude and elevation regions in western China under future climate conditions, particularly under high‐emission scenarios.

## 4 Discussion

In China, *A. pungens* was historically considered a weak invasive species compared to its exotic congener *A. philoxeroides* , due to its short invasion history, low growth rate, restricted distribution, and low economic impacts in China (Wang et al. 2010). Hence, knowledge on the distribution of this species was not well documented. The current study shows that *A. pungens* has the potential to spread significantly further than its current known distribution under current and future climatic conditions and with human activity, such as transportation, agriculture and construction. The MaxEnt model also allows for jurisdictions to plan early warning and/or preventative measures to reduce the possibility of *A. pungens* becoming established.

In this study, the MaxEnt model exhibited high predictive performance, with AUC and TSS values indicating strong discrimination ability and consistency between predicted and observed suitability (Sun et al. 2020; Bai et al. 2022). This level of model reliability provides a solid basis for interpreting the predicted distribution patterns of *A. pungens* and for discussing its potential invasion risk and management implications under current and future climate scenarios. To further evaluate the reliability of model predictions, we compared the predicted suitability classes under current climatic conditions with field occurrence data of *A. pungens* . Among the 372 field survey records, 212 points (57.0%) were located in highly suitable areas and 128 points (34.4%) in moderately suitable areas, accounting for 91.4% of all occurrences. In contrast, only 29 records (7.8%) fell within slightly suitable areas, and merely 3 records (0.8%) were located in unsuitable areas. This strong spatial concordance between model predictions and field observations indicates that the model effectively captures the current distribution pattern of *A. pungens* , supporting the robustness and ecological reliability of the predicted suitability maps.

This study found that 5.6% of China's land area, encompassing approximately 53.66 × 10^4^ km^2^, provides suitable habitat for *A. pungens* under the current climate scenarios. The potential suitable areas of *A. pungens* are primarily concentrated in southwestern China, especially in Yunnan Province and southern Sichuan Province. This spatial pattern is consistent with the region's warm, humid climate and high levels of anthropogenic disturbance (Chen et al. 2022; Li et al. 2022), which are known to favor the establishment and spread of invasive species (Liu et al. 2024; Xie, Huang, Xie, et al. 2024). These distribution characteristics are partially aligned with the actual field observations.

From field investigations, *A. pungens* has a broad distribution and occurred in most areas of Yunnan Province. However, the population outbreak and serious harm of *A. pungens* are mainly concentrated in the dry and hot valleys, which may be caused by both local climate environments and ecological habits of *A. pungens* . The dry and hot valleys are generally characterized by drought, low rainfall, intense solar radiation, and poor soil fertility, which is not conducive to the seed germination, growth and biomass accumulation of surrounding plant species under natural conditions. In contrast, *A. pungens* thrives in this environment, a resilience attributed to its robust stem and deep taproot system. It was found that *A. pungens* was strongly associated with highways, villages, towns, and communities, suggesting its invasion and spread are largely driven by transportation networks and anthropogenic disturbances. In addition, the numerous leaf spiny burrs and fruit spines of *A. pungens* , readily adhere to rubber soled shoes or tyres, facilitating its long‐distance dispersal to new areas (Parsons and Cuthbertson 2001). These field observations are consistent with the modeling results, which identified temperature seasonality (Bio4) as the dominant factor shaping the distribution of *A. pungens* . Dry and hot valleys in Yunnan are typically characterized by moderate temperature seasonality, with Bio4 values largely falling within the optimal range identified by the response curves. Although these environments are dry, the relatively low contribution of precipitation variables (Bio12 and Bio17) suggests that water availability is not the primary limiting factor for *A. pungens* , which appears to be more strongly constrained by thermal conditions than by precipitation.

With global climate warming, most plant and animal species are expected to shift their ranges towards higher latitudes and elevations (Fang et al. 2018). Consistent with this trend, our results show that under future climate scenarios, the suitable areas of *A. pungens* in China are projected to expand from its core area in the southwest towards the northwest. This indicates that *A. pungens* possesses a high degree of climatic adaptability, enabling it to expand into new regions under changing environmental conditions (Clements and Ditommaso 2011; Colautti and Barrett 2013; Bradley et al. 2024). Field surveys found that *A. pungens* occurred in a wide range of elevational range from 650 m (Mengyang Township, Jinghong City) to 3600 m (Benzilan Township, Deqin County) in Yunnan Province. This wide distribution demonstrates a high risk of further expansion into new areas at higher latitudes and elevations, particularly under the combined pressures of increased human disturbance and climatic change.

The study found an increase in the suitable habitats for *A. pungens* under future climate scenarios. This trend is consistent across all future climatic change, with a significant expansion projected by the end of the century under the worst climate scenario (ssp585), where suitable habitats are expected to increase by 41.4%. Notably, certain suitable areas remain consistently present and geographically stable across all scenarios, suggesting strong climatic resilience. These results show that the targeted research and management efforts for *A. pungen* not only need to pay attention to the current and future suitable habitats, but also mitigate its negative impacts in invaded areas.

Environmental variable analysis revealed that temperature seasonality (Bio4) was the most important factor influencing the distribution of *A. pungens* . However, the potential distribution of its exotic congener *A. philoxeroides* was mostly affected by both temperature and precipitation (Yan et al. 2020). *Alternanthera pungens* exhibited high suitability when temperature seasonality values ranged from approximately 428 to 478, corresponding to a monthly temperature standard deviation of 4.28°C–4.78°C. This indicates a preference for regions with moderate seasonal temperature variability. Ecologically, moderate temperature seasonality may shape the distribution of *A. pungens* by allowing sustained growth during warm periods while avoiding excessive thermal stress in colder seasons. The importance of the mean temperature of the coldest quarter further suggests that winter thermal conditions constrain survival and regrowth, particularly for a species adapted to warm and dry environments. Suitability declined outside this range, suggesting that *A. pungens* may be less adapted to both climatically uniform and highly fluctuating environments, as inferred from the response curve.

Similar patterns have been observed in other subtropical invasive species, such as *Parthenium hysterophorus* L. (Asteraceae) (Liu et al. 2023), *Reynoutria japonica* Houtt. (Polygonaceae) (Miroshnyk et al. 2025), and *Prosopis* spp. (Fabaceae) and *Acacia mearnsii* De Wild. (Fabaceae) (Mengistu et al. 2023), where temperature seasonality plays a critical role in shaping ecological niches and constraining range limits. Wang et al. (2010) reported that *A. pungens* produced more biomass with low availability of water and nutrients in comparison with *A. philoxeroides* and could potentially exhibit stronger invasiveness than *A. philoxeroides* if it spreads to relatively arid areas in western China. These were consistent with our field findings that the population outbreak and serious harm of *A. pungens* are mainly concentrated in the dry and hot valleys, whereas *A. philoxeroides* is an aquatic or semi‐aquatic species. It was found that *A. philoxeroides* is mainly constrained to humid natural habitats (e.g., rivers, lakes and marshes) in the dry and hot valleys from the field surveys, but *A. pungens* is widely distributed in other habitats, which show that *A. philoxeroides* and *A. pungens* have different ecological adaption and invasion mechanisms under dry and hot natural conditions.

The timely and accurate assessment of suitable habitats for invasive species is crucial to provide early warning and decision‐making reference for the prevention and control of non‐native species (Li et al. 2023). In this study, the MaxEnt model was used to predict the potential suitable areas of *A. pungens* under current and future climate scenarios in China. The results indicated that the potential suitable habitats for *A. pungens* are currently concentrated in southwestern China and are projected to expand under all future climate scenarios. Based on the spatial patterns of highly suitable areas, priority monitoring of *A. pungens* should be given to central Yunnan (e.g., Dali, Lijiang, and Chuxiong) and southern Sichuan (e.g., Panzhihua), where highly suitable habitats are currently concentrated. Under future climate scenarios, increased attention is also warranted for surrounding regions, including Baoshan, Lincang, Puer, Yuxi, Kunming, Honghe (Yunnan), and Liangshan (Sichuan), which show progressive expansion of suitable areas. In addition, the centroid of suitable habitats is projected to shift northwestward within Yunnan Province under most future scenarios, generally moving from the Kunming–central Yunnan region towards Chuxiong and the Lijiang area.

This directional shift of *A. pungens* suggests that major transportation corridors connecting central Yunnan with western and northwestern Yunnan, as well as corridors linking Yunnan with southern Sichuan, may serve as potential pathways for human‐mediated dispersal. Enhanced quarantine, roadside monitoring, and early detection efforts are therefore recommended along these key transportation corridors to reduce the risk of further spread. Accordingly, integrated management strategies (e.g., agricultural, physical, chemical, and ecological controls) should be implemented promptly in climatically suitable regions. Future efforts should also prioritize strengthening early warning and monitoring systems and raising awareness among farmers and land managers to improve early identification and containment of *A. pungens* before its establishment in newly suitable areas.

## 5 Conclusion

Predicting the potential distribution of invasive plants under climate change and exploring their ecological impacts are important for invasive species management and biodiversity conservation. Our results showed that the MaxEnt model fit (area under the curve value = 0.97 and true skill statistic value = 0.910) was robust and the most important environmental variables affecting the potential suitability of a habitat included temperature seasonality and mean temperature of coldest quarter. The suitable area of *A. pungens* is mainly located in southern China under the current climate scenarios, and the suitable areas for *A. pungens* in China will expand northward, with a maximum projected growth rate of 41.4% in the 2070 s under future climate scenarios. To gain a more nuanced understanding of the distribution patterns and facilitate the application of our findings in conservation and management efforts, some biologically important factors (e.g., transportation, human activities) and field studies should be integrated and validated.

## Author Contributions

**Fengping Zheng:** conceptualization (equal), data curation (equal), supervision (equal). **Wei Zhang:** data curation (equal), formal analysis (equal), methodology (equal), visualization (equal), writing – original draft (equal). **Qiurui Li:** data curation (equal), investigation (equal), writing – original draft (equal). **Zhijie Wang:** investigation (equal). **Gaofeng Xu:** investigation (equal). **David Roy Clements:** conceptualization (equal), writing – review and editing (equal). **Bin Yao:** investigation (equal). **Guimei Jin:** investigation (equal). **Shaosong Yang:** investigation (equal). **Shicai Shen:** conceptualization (equal), data curation (equal), funding acquisition (equal), investigation (equal), project administration (equal), supervision (equal), writing – original draft (equal). **Fudou Zhang:** conceptualization (equal), funding acquisition (equal), project administration (equal). **Michael Denny Day:** writing – review and editing (equal).

## Funding

This research was supported by the National Key Research and Development Program of China (2024YFC2607600), Yunnan Fundamental Research Project (202501AS070027), Yunnan Provincial Agricultural Basic Research Joint Special Project (202401BD070001‐019), National Natural Science Foundation of China (31960569), Key Research and Development Program of Yunnan Province (202103AF140007, 202203AE140008), and Ten Thousand Talent Program (Young Top‐Notch Talent) of Yunnan Province (YNWR‐QNBJ‐2018‐201).

## Conflicts of Interest

The authors declare no conflicts of interest.

## Supporting information


**Data S1:** ece373124‐sup‐0001‐Supplementary_Materials.xls.

## Data Availability

All the required data are uploaded as Supporting Information.
